# D614G Substitution of SARS-CoV-2 Spike Protein Increases Syncytium Formation and Virus Titer via Enhanced Furin-Mediated Spike Cleavage

**DOI:** 10.1128/mBio.00587-21

**Published:** 2021-07-27

**Authors:** Ya-Wen Cheng, Tai-Ling Chao, Chiao-Ling Li, Sheng-Han Wang, Han-Chieh Kao, Ya-Min Tsai, Hurng-Yi Wang, Chi-Ling Hsieh, You-Yu Lin, Pei-Jer Chen, Sui-Yuan Chang, Shiou-Hwei Yeh

**Affiliations:** a Department of Microbiology, National Taiwan University College of Medicine, Taipei, Taiwan; b Department of Clinical Laboratory Sciences and Medical Biotechnology, National Taiwan University College of Medicine, Taipei, Taiwan; c Hepatitis Research Center, National Taiwan University Hospital, Taipei, Taiwan; d Graduate Institute of Clinical Medicine, National Taiwan University College of Medicine, Taipei, Taiwan; e Department of Internal Medicine, National Taiwan University Hospital, Taipei, Taiwan; f National Taiwan University Center for Genomic Medicine, National Taiwan University College of Medicine, Taipei, Taiwan; g Department of Laboratory Medicine, National Taiwan University Hospital, Taipei, Taiwan; Virginia Polytechnic Institute and State University

**Keywords:** furin, SARS-CoV-2, spike, syncytium

## Abstract

Since the D614G substitution in the spike (S) protein of severe acute respiratory syndrome coronavirus 2 (SARS-CoV-2) emerged, the variant strain has undergone a rapid expansion to become the most abundant strain worldwide. Therefore, this substitution may provide an advantage for viral spreading. To explore the mechanism, we analyzed 18 viral isolates containing S proteins with either G614 or D614 (S-G614 and S-D614, respectively). The plaque assay showed a significantly higher virus titer in S-G614 than in S-D614 isolates. We further found increased cleavage of the S protein at the furin substrate site, a key event that promotes syncytium formation, in S-G614 isolates. The enhancement of the D614G substitution in the cleavage of the S protein and in syncytium formation has been validated in cells expressing S protein. The effect on the syncytium was abolished by furin inhibitor treatment and mutation of the furin cleavage site, suggesting its dependence on cleavage by furin. Our study pointed to the impact of the D614G substitution on syncytium formation through enhanced furin-mediated S cleavage, which might increase the transmissibility and infectivity of SARS-CoV-2 strains containing S-G614.

## INTRODUCTION

The severe acute respiratory syndrome coronavirus 2 (SARS-CoV-2) infection causes a rapid accumulation of confirmed and fatal cases and poses a threat to public health around the world. A better understanding of the virus’s evolution and characterization of viral genetic variations usually provide valuable insights into the mechanisms linked to pathogenesis, antiviral drug resistance, and immune responses, which also impact the development of new vaccines, antiviral drugs, and diagnostic tests (1–3). Therefore, analysis of viral genomes and monitoring of the evolutionary trajectory of SARS-CoV-2 over time have been meticulously conducted to identify any specific genetic variations that contribute to the transmissibility and virulence of SARS-CoV-2 (4–6).

Among the genetic variations that have evolved during the course of the SARS-CoV-2 outbreak, the D614G substitution in the spike (S) protein, which corresponds to a change of the A nucleotide at genome position 23403 to a G, has been identified as the signature of the A2a clade of SARS-CoV-2, the most prevalent clade (7, 8). This substitution emerged at low frequency in early March 2020 but rapidly expanded to become the most abundant clade worldwide by April and May 2020 (7), and it was thus proposed to provide a selective fitness advantage during the outbreak. B. Korber et al. recently reported that the strain containing the spike protein with G at position 614 (S-G614) is associated with higher viral loads in the upper respiratory tracts of infected individuals (though not increased disease severity) than the loads produced by viruses containing S-D614 (7). Several recent reports further demonstrated that pseudotyped viruses or the engineered viruses containing S-G614 exhibit significantly higher infectivity than those containing S-D614 (9–11). Sera from most convalescent CoV disease 2019 (COVID-19) patients may neutralize both S-D614- and S-G614-pseudotyped viruses with comparable efficiencies (7, 12). As this mutation locates outside the receptor binding domain (RBD) of S, similar affinities for binding of S-D614 and S-G614 to the angiotensin-converting enzyme 2 (ACE2) receptor were documented (13–15). These findings thus raised the possibility that the D614G substitution confers increased infectivity and transmissibility neither through an increased binding to ACE2 nor through increased escape of immune surveillance. Elucidating the molecular basis for the higher infectivity of the D614G virus is urgent to understand its predominance and to design an effective treatment strategy for patients.

**FIG 1:**
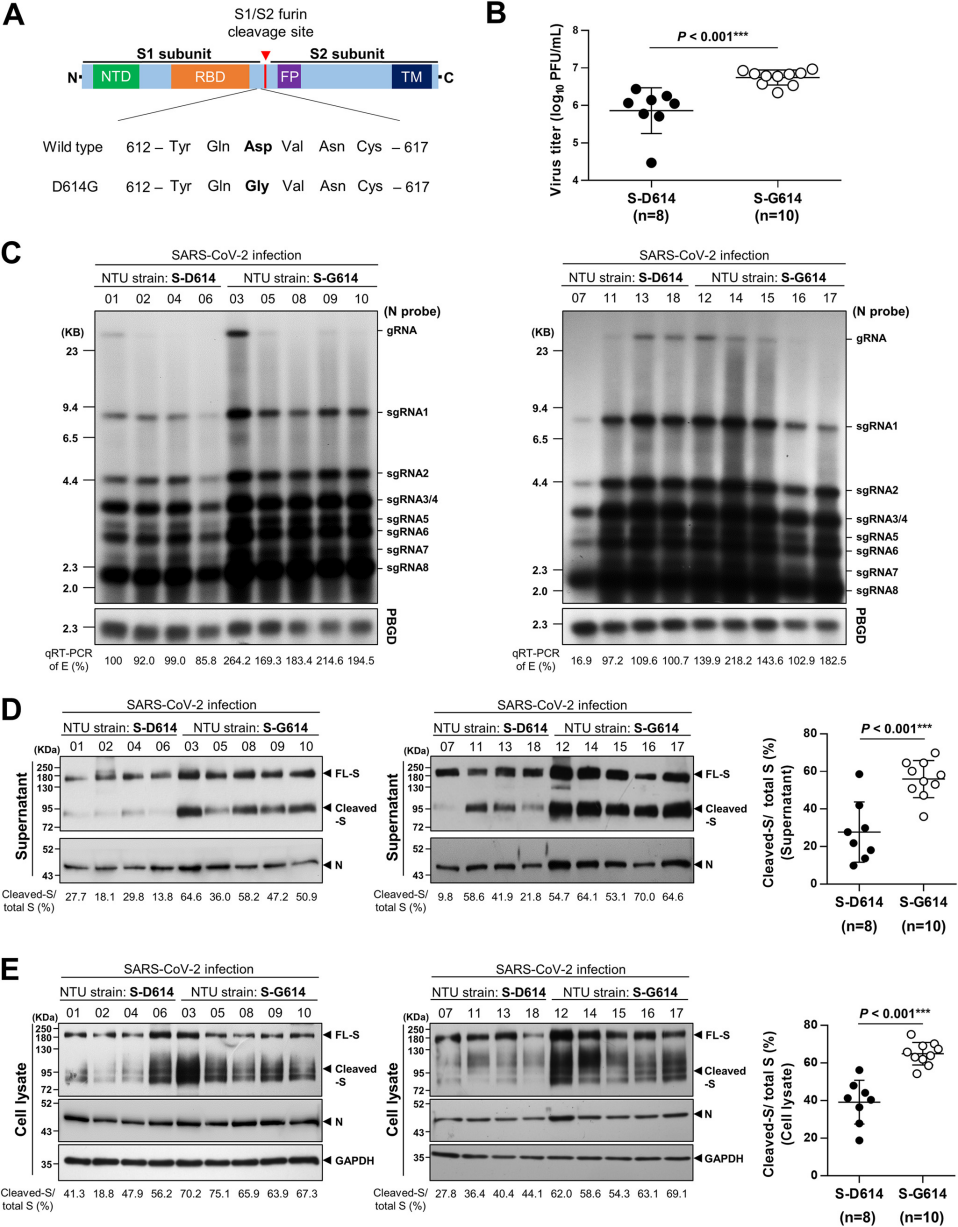
D614G substitution in the spike (S) protein enhanced viral production and increased cleavage of the S protein compared to that in wild-type spike in Calu-3 cells infected with SARS-CoV-2 isolates. (A, top) Schematic illustration of the SARS-CoV-2 spike protein, including the N-terminal domain (NTD), receptor binding domain (RBD), fusion peptide (FP), and transmembrane domain (TM). The S1/S2 subunits were separated by a furin cleavage site, indicated by the red arrowhead. (Bottom) Sequences of aa 612 to 617 in the wild-type and mutant D614G spike proteins. The 614-amino-acid Asp (D)-to-Gly (G) substitution in the spike protein is marked in bold. (B) Comparison of the virus titers (PFU per milliliter) (MOI = 0.5) in the supernatants of Calu-3 cells infected with SARS-CoV-2 strains NTU01 to NTU18 (8 strains expressing wild-type S-D614 and 10 strains expressing mutant S-G614) at 24 h postinfection. Data are means ± SD (*P < *0.001***). (C) Northern blot analysis of viral RNA isolated from Calu-3 cells infected with SARS-CoV-2 strains NTU01 to NTU18 at 24 h postinfection. Viral RNA was quantified by qRT-PCR targeting the E gene, as indicated below the Northern blot results. The percentage for the E gene was normalized to that of NTU01 (100%). gRNA, genomic RNA; sgRNA, subgenomic RNA. (D, left) Immunoblot of S protein extracted from the supernatants of S-D614-containing (lanes 1 to 4) or S-G614-containing (lanes 5 to 9) virus-infected Calu-3 cells at 24 h postinfection (NTU01 to 18; MOI = 0.5). Full-length (FL) S proteins, cleaved S proteins, and nucleocapsid (N) proteins are marked as indicated. The percentage of cleaved S to total (cleaved plus full-length) S protein for each SARS-CoV-2 strain is indicated at the bottom of the immunoblot results. The percentages in the S-D614- and S-G614-containing viruses were compared and are presented as means ± SD in the right panel (*P < *0.001***). (E, left) Immunoblot of S protein extracted from the cell lysates as described for panel D. The percentage of cleaved S to total (cleaved plus full-length) S protein for each SARS-CoV-2 strain (NTU01 to 18) is indicated below the immunoblot results. The percentages were compared and are presented as means ± SD in the right panel (*P < *0.001***).

The host protease-mediated cleavage of envelope protein is involved in the maturation, pathogenesis, and infectivity of many viruses. S of SARS-CoV-2 also undergoes two proteolytic cleavages critical for membrane fusion and viral entry. One cleavage site is located at the S1 and S2 boundary, a polybasic motif recognized by furin and related proprotein convertases (PCs); the other is located at the S2′ site within the S2 domain, which is recognized by the TMPRSS2 protease (16). In our recent study, we found that the cleavage of S by furin/PCs at the S1/S2 boundary is critical for S-mediated syncytium formation, another pathogenic event that contributes to increased viral transmission (17). As noted previously, the D614G substitution is located in the C-terminal region of the S1 domain of the S protein, close to the furin cleavage site (between amino acids [aa] 685 and 686) (Fig. 1A). Meanwhile, recent cryogenic electron microscopy (cryo-EM) studies suggested that the D614G substitution might induce conformational changes in the S protein (13, 18, 19). It thus raised the possibility that the D614G substitution contributes to the increased accessibility of the S protein for cleavage by furin through a conformational change and thus increases the membrane fusion activity of the virus; this may be the basis for the increased infectivity, transmission capability, and virus titer of S-G614-containing SARS-CoV-2. To test this hypothesis, we first compared the virus titers and rates of cleavage of the S protein of 18 clinical SARS-CoV-2 isolates. The effects of the D614G substitution on enhanced S cleavage and syncytium formation were further quantitatively validated in cells expressing S protein. This cell-based assay system was further used to examine the critical role of furin-mediated S cleavage for the effect of the D614G substitution. We expect that the findings will provide an explanation for the rapid expansion of S-G614-containing SARS-CoV-2 and help develop a therapeutic strategy for intervening in its spread in the population.

## RESULTS

### The virus titer was significantly higher in S-G614-containing viral isolates than in S-D614-containing viral isolates.

To investigate the effect of the D614G mutation on SARS-CoV-2 replication, we first compared the virus titers of 18 clinical SARS-CoV-2 isolates (NTU01 to NTU18) (see Tables S1 and S2 in the supplemental material) containing either S-D614 or S-G614 from infected Calu-3 and Vero E6 cells. Interestingly, we found that the S-G614-containing viruses (*n* = 10) had significantly higher virus titers than the S-D614-containing viruses (*n* = 8), as determined by a plaque assay (Fig. 1B [Calu-3 cells]; Fig. S1A, [Vero E6 cells]). Consistently, Northern blotting and quantitative reverse transcription-PCR (qRT-PCR) analysis confirmed the higher viral RNA levels in cells infected with S-G614-containing viruses than in cells infected with S-D614-containing viruses (Fig. 1C [Calu-3 cells; *P < *0.001]; Fig. S1B [Vero E6 cells; *P = *0.0164]). The results obtained with the virus isolates suggest a possible functional effect of the D614G substitution in increasing virus production.

10.1128/mBio.00587-21.1 FIG S1 Virus production was higher for S-G614-containing SARS-CoV-2 than for S-D614-containing SARS-CoV-2, which was associated with an increase in the cleavage of the S protein in Vero E6 cells. (A) Comparison of the virus titers (PFU per milliliter) (MOI = 0.01) in the supernatants of Vero E6 cells infected with SARS-CoV-2 strains NTU01 to NTU18 (8 strains expressing wild-type S-D614 and 10 strains expressing mutant S-G614) at 24 h (left) and 48 h (right) postinfection. Data are means ± SD (*P < *0.05*; *P < *0.01**). (B) Northern blot analysis of viral RNA isolated from Vero E6 cells infected with SARS-CoV-2 strains (NTU01 to NTU18) at 48 h postinfection. Viral RNA was quantified by qRT-PCR targeting the E gene, as indicated below the Northern blot results. The ratio (percentage) of the E gene was normalized to the NTU01 level (100%). (C, left) Representative immunoblot of S protein extracted from the supernatants and cell lysates of S-D614-containing (lanes 1 to 4) or S-G614-containing (lanes 5 to 9) virus-infected Vero E6 cells at 48 h postinfection (MOI = 0.01). Full-length (FL) S proteins, cleaved S proteins, and nucleocapsid (N) proteins are marked as indicated. The ratio of cleaved S to full-length S protein for each SARS-CoV-2 strain is indicated below the immunoblot results. The ratios in the S-D614- and S-G614-containing viruses were compared and are presented as means ± SD in the right panel (*P < *0.05*). (D, left) Immunoblot of S protein extracted from the cell lysates as described for panel C. The ratio of cleaved S to full-length S protein for each SARS-CoV-2 strain is indicated below the immunoblot results. The ratios were compared and are presented as means ± SD in the right panel (*P < *0.05*). Download FIG S1, TIF file, 4.0 MB. Copyright © 2021 Cheng et al. 2021 Cheng et al. https://creativecommons.org/licenses/by/4.0/ This content is distributed under the terms of the Creative Commons Attribution 4.0 International license.

10.1128/mBio.00587-21.3 TABLE S1 SARS-CoV-2 genome (GISAID ID). The sequencing data and the accession codes of 18 virus isolates were deposited in GISAID (https://www.gisaid.org/CoV2020/). Download Table S1, DOCX file, 0.02 MB. Copyright © 2021 Cheng et al. 2021 Cheng et al. https://creativecommons.org/licenses/by/4.0/ This content is distributed under the terms of the Creative Commons Attribution 4.0 International license.

10.1128/mBio.00587-21.4 TABLE S2 Summary of the mutations in SARS-CoV-2 isolates NTU01 to NTU18. SARS-CoV-2 genetic sequences, including NTU01 to NTU18 and the reference sequence (MN_908947), were aligned and compared. Nucleotide 23403, corresponding to aa 614 of the spike protein, is marked in red text, and the orange-shaded nucleotides indicate nucleotides different from the reference nucleotides. Sequence data were obtained from GISAID (https://www.gisaid.org/CoV2020/); the GISAID accession numbers for these 18 isolates are listed in Table S1. Download Table S2, DOCX file, 0.04 MB. Copyright © 2021 Cheng et al. 2021 Cheng et al. https://creativecommons.org/licenses/by/4.0/ This content is distributed under the terms of the Creative Commons Attribution 4.0 International license.

### S-G614-containing viral isolates showed increased cleavage of the S protein compared to that of S-D614-containing viral isolates.

To examine if the D614G substitution might contribute to increased accessibility of the S protein for cleavage by furin, we compared the patterns of S protein cleavage at this site for viruses containing either the S-G614 or the S-D614 protein. Viruses in the supernatants of cells infected with either group of viruses were harvested for immunoblot analysis. Interestingly, the results showed significantly increased cleavage of the S protein into the S1 and S2 fragments in S-G614-containing viruses compared to that in S-D614-containing viruses in Calu-3 cells (Fig. 1D); the cleaved S/total S percentage was shown to be 55.95% ± 9.86% (in S-G614 viruses) versus 27.71% ± 16.05% (in S-D614 viruses) (*P < *0.001) (Fig. 1D, right). Moreover, we found a higher ratio of spike to nucleocapsid (N) in the S-G614- than in the S-D614-containing viruses (total S/N, 1.9 ± 0.44 versus 1.0 ± 0.31; *P < *0.001; cleaved S/N, 3.7 ± 0.39 versus 1.0 ± 0.27; *P < *0.001) (Fig. S2).

10.1128/mBio.00587-21.2 FIG S2 The amount of S protein was significantly higher in S-G614-containing than in S-D614-containing SARS-CoV-2 viruses. The ratios of total (cleaved plus full-length) S/N (left) and cleaved S/N (right) for each SARS-CoV-2 isolate were derived from the immunoblot results for the supernatants of individual virus-infected Calu-3 cells, as shown in Fig. 1D, and are presented as means ± SD (*P < *0.001***). S, spike; N, nucleocapsid. Download FIG S2, TIF file, 1.1 MB. Copyright © 2021 Cheng et al. 2021 Cheng et al. https://creativecommons.org/licenses/by/4.0/ This content is distributed under the terms of the Creative Commons Attribution 4.0 International license.

Consistently, a significant difference in the rates of cleavage of the S protein between the lysates from cells infected with the two groups of viruses was also demonstrated by immunoblotting (Fig. 1E), with the cleaved S/total S percentages being 64.96% ± 5.99% in S-G614 viruses versus 39.13% ± 11.65% in S-D614 viruses (*P < *0.001) (Fig. 1E, right). A higher cleavage of S in S-G614- than in S-D614-containing viruses was also supported by the results from Vero E6 cells infected with the two groups of viruses (Fig. S1C and D). Therefore, these results suggested a putative effect of the D614G substitution in enhancing the cleavage of the S protein.

### The syncytial phenotype and S cleavage were increased in S-G614-expressing cells compared to those in S-D614-expressing cells.

**FIG 2:**
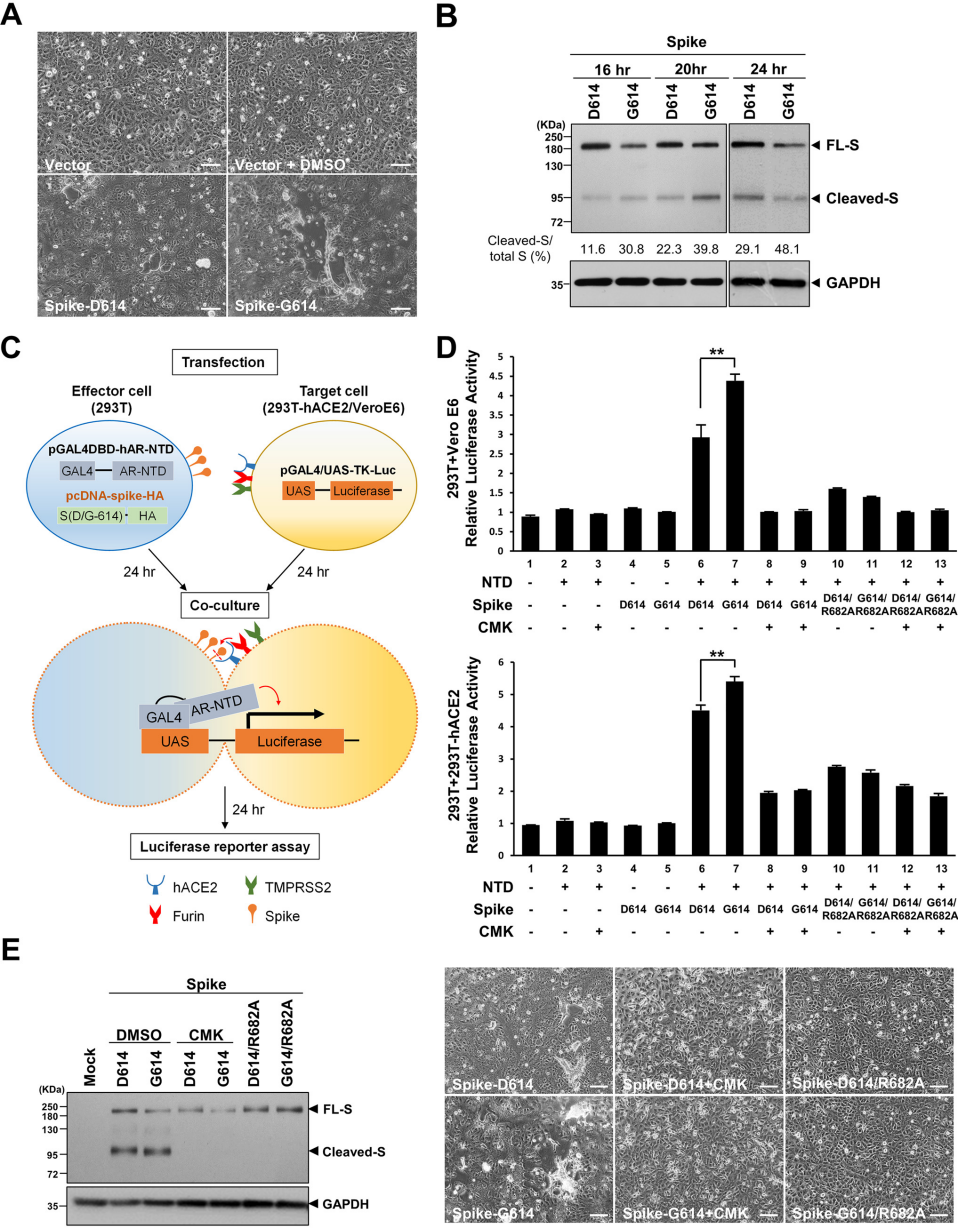
The syncytium formation and fusion activity of the SARS-CoV-2 S protein was higher in S-G614-expressing cells than in S-D614-expressing cells and dependent on furin/PC-mediated cleavage of S protein. (A) Microscopic observation of syncytia in Vero E6 cells expressing the S-D614 or S-G614 protein. Scale bars, 100 μm. (B) Immunoblot analysis of lysates from Vero E6 cells transfected with expression constructs for S-D614 or S-G614 harvested at 16 to 24 h posttransfection. The immunoblot was probed with anti-S antibody, and the full-length (FL) and cleaved S proteins are marked as indicated; GAPDH was included as a loading control. The percentage of cleaved S versus total (cleaved plus full-length) S is indicated below the immunoblot results. (C) Schematic illustration of the one-hybrid luciferase reporter assay designed to quantitatively evaluate the fusion activity induced by SARS-CoV-2 S protein. Effector 293T cells were cotransfected with an expression plasmid for S-D614 or S-G614 and the pGAL4DBD-hAR-NTD plasmid. The target 293T-hACE2 or Vero E6 cells were transfected with pGAL4/UAS-TK-Luc. At 24 h posttransfection, the effector and target cells were cocultured for 24 h and harvested to assay the luciferase activity. (D) Representative results of the luciferase reporter assay showing luciferase activity induced by S-D614 or S-G614 protein (upper panel, in Vero E6 target cells; lower panel, in 293T-hACE2 target cells). The luciferase activity in cells expressing the SARS-CoV-2 S-D614/S-G614 protein or D614/R682A or G614/R682A protein with or without treatment with a furin/PC inhibitor (CMK, 50 μM) was assessed as indicated. The results were derived from three independent experiments and are shown as means ± SD (**, *P < *0.01). (E) Effects of treatment with CMK (50 μM) and the R682A mutation of S on syncytium formation induced by S-D614 or S-G614 protein. (Left) Immunoblot analysis of the lysates from Vero E6 cells transfected with the indicated plasmids with or without CMK treatment; (right) microscopic observation of cell morphology in Vero E6 cells transfected with the indicated plasmids with or without CMK treatment. Scale bars, 100 μm.

Genetic heterogeneity beyond the D614G substitution among different SARS-CoV-2 isolates might cause confusion regarding our observation. Therefore, we tested our hypothesis in cultured cells expressing only the S protein. Codon-optimized expression plasmids for wild-type S-D614 and the single-substitution mutant (S-G614) were individually transfected into Vero E6 cells, which were harvested at 24 h posttransfection for analysis. We first examined the syncytial phenotype via the observation of fused cells containing multiple nuclei visible by light microscopy. Compared with control cells transfected with only the vector, cells expressing the S-D614 protein exhibited a moderate syncytial phenotype, which was increased in cells expressing the mutant S-G614 protein (Fig. 2A). Immunoblot analysis further revealed increased cleavage of the S-G614 proteins relative to the S-D614 protein and a higher cleaved S/total S ratio in the lysate of S-G614-expressing cells than in S-D614-expressing cells, not only at 24 h posttransfection but also at 16 and 20 h posttransfection (30.8% versus 11.6%, 39.8% versus 22.3%, and 48.1% versus 29.1% at 16, 20, and 24 h posttransfection, respectively) (Fig. 2B). These results are consistent with the findings from the virus infection system shown in Fig. 1E.

### S-mediated syncytium formation was increased in S-G614-expressing cells compared to that in S-D614-expressing cells, dependent on furin-mediated S cleavage.

To confirm the effect of the D614G substitution on S and ACE2 binding-mediated cell syncytium formation, we established a luciferase-based reporter assay to quantitatively compare levels of syncytium induction by the S-G614 and S-D614 proteins (schematically illustrated in Fig. 2C). As documented, the interaction between S and ACE2 at the membranes of infected cells and adjacent cells primes the cleavage of S by host proteases to release the fusion peptide essential for syncytium formation (20). Therefore, in our experimental design, we selected ACE2-null 293T cells transfected with an expression plasmid for either S-D614 or S-G614 as S^+^ and ACE2^–^ effector cells. Two ACE2^+^ target cell lines were selected: 293T cells stably transfected with human ACE2 (293T-hACE2) and Vero E6 cells (with endogenous ACE2 expression). Neither effector cells nor target cells alone developed syncytia due to the lack of the binding partner (ACE2 or the S protein). To quantitatively examine the fusion of effector and target cells, we developed a one-hybrid luciferase reporter assay. The pGAL4DBD-hAR-NTD plasmid was cotransfected with an individual S expression plasmid into the effector cells; the pGAL4/UAS-TK-Luc plasmid was transfected into the target cells. The effector and target cells were then cocultured and harvested for reporter activity analysis. The expression of pGAL4/UAS-TK-Luc in the target cells was activated only upon formation of syncytia consisting of effector and target cells (mediated by the interaction of S and ACE2), which is driven by the transcriptional activator encoded by the pGAL4DBD-hAR-NTD plasmid in the effector cells.

As expected, the expression of pGAL4DBD-hAR-NTD or S alone in the effector cells did not activate luciferase expression in the target cells (Fig. 2D, lanes 2 to 5), which was elevated only when pGAL4DBD-hAR-NTD and the S protein were coexpressed in the effector cells. The expression of S-G614 significantly increased the luciferase activity compared to that upon the expression of S-D614 in the effector cells (Fig. 2D, lane 6 versus lane 7). The results from the reporter assay thus provide quantitative evidence supporting increased syncytium induction by the mutant S-G614 protein compared with that induced by the S-D614 protein, consistent with the findings from light microscopy observation.

Since our previous study showed that furin inhibitors can block spike cleavage and spike-mediated syncytium formation both in SARS-CoV-2-infected cells and spike-expressing cells (17), we want to further examine whether the enhanced syncytium formation by S-G614 expression was mediated through cleavage by furin/PC protease(s). We treated S-expressing effector cells with the furin/PC inhibitor decanoyl-RVKR-chloromethylketone (CMK). Immunoblot analysis demonstrated that cleavage of the S-D614 or S-G614 protein was completely blocked by CMK treatment (Fig. 2E, left). The syncytial phenotype in the S-D614- and S-G614-expressing cells was also significantly decreased by CMK treatment (Fig. 2E, right). The difference in luciferase activity induced by the S-D614 and S-G614 proteins was diminished by CMK treatment (Fig. 2D, lane 8 versus lane 9), suggesting that the enhanced syncytium formation induced by the S-G614 protein is dependent on the presence of active furin/PCs in cells. Consistently with this finding, the difference in the effects of S-D614 and S-G614 on reporter activity was abolished when the furin substrate site was mutated by introduction of the R682A substitution (Fig. 2D, lane 10 versus lane 11); the effects of the substitution on the cleavage of the S protein and syncytium formation are shown in Fig. 2E. All together, these results suggest that the putative function of the D614G mutation in the S protein of SARS-CoV-2 is dependent on enhanced cleavage at the furin substrate motif at the S1/S2 boundary, which contributes to an increased membrane fusion activity.

## DISCUSSION

Recently, several studies showed that the D614G variant SARS-CoV-2 replicates more efficiently in primary human proximal airway epithelial cells than the wild-type virus. They also provided *in vivo* evidence showing that the D614G variant exhibited significantly faster droplet transmission between hamsters than the wild-type virus at early stages after infection (9, 11). Our study here provides a possible mechanistic explanation in support of the notion that the D614G substitution can increase virus transmission through enhanced membrane fusion mediated by increased cleavage of the S protein by furin/PC proteases.

In our previous study, we examined the effect of furin cleavage on viral replication by either preinfection treatment or postinfection treatment of the virus-infected cells with the CMK furin inhibitor (17). The results suggested that the furin-mediated cleavage of spike might not contribute to viral RNA synthesis and virion release but had a critical role in membrane fusion for the viral entry step. The current study further revealed that the D614G substitution in S might contribute to increase the cleavage by furin/PCs and thus increase the syncytium formation of S-G614-containing SARS-CoV-2 strains. Therefore, the effect of enhanced furin cleavage of the spike protein caused by the D614G substitution might increase viral entry into the cells and contribute to higher virus titers after multiple infection cycles.

Regarding the mechanism, as revealed by cryo-EM, Wrobel et al. demonstrated that proteolytic cleavage of spike at the S1/S2 boundary could turn the spike trimer from a “closed” form into an “open” form, a receptor-binding-competent structure with high affinity for the ACE2 receptor (21). Park et al. also showed that cleavage of S by furin might prime for subsequent S cleavage by TMPRSS2 and help release the fusion peptide for subsequent membrane fusion activity (22). These studies thus provide possible mechanisms for increased furin-mediated S cleavage in S-G614 viruses to enhance viral entry, which meanwhile may also contribute to higher syncytium formation of cells infected with S-G614 viruses. Both events may contribute to the increase in virus titer of the S-G614 viruses. In fact, the contribution of increased syncytia in the spreading of viruses has been documented for several viruses (22–26), possibly through direct cell-to-cell spreading and escape from the innate immune surveillance (27, 28). Based on these observations, the increased syncytium formation induced by the D614G substitution might increase the spreading of SARS-CoV-2. This possibility is worthy of being tested *in vivo* in proper animal models.

Regarding how the substitution may affect furin cleavage, through cryo-EM analysis, several studies well demonstrated the effects of the D614G substitution on the structure of spike protein. As noted, the RBDs in the D614 spike trimer are presented mainly as the closed form (13, 18, 19). One structure change caused by the D614G substitution is the loss of the salt bridge between D614 and K854, which reduced the stability of the spike trimer in the closed form. Therefore, the RBDs in the G614 spike trimer are preferentially presented in the open form, which is more competent for ACE2 binding. Meanwhile, disruption of the salt bridge between D614 and K854 might unfold a domain (aa 827 to 855) in the S2 subunit; this unfolding promotes fusion-peptide-containing domain (aa 815 to 825) to undergo membrane fusion (19, 21). These structural changes caused by the D614G substitution might contribute to a higher affinity of the spike protein for the furin/PCs, which has been demonstrated by the computational prediction of furin binding to the RRAR motif of S-G614 versus S-D614 and is preliminarily supported by the results of an *in vitro* proteolytic assay (18, 29).

Though the mechanism for the increased cleavage by furin/PCs by the D614G substitution was delineated in assays of cells expressing only the S protein, it has been well supported by virus-infected cells, as most S-G614-containing viruses show more evidence of the cleavage of S in association with higher virus titers. Despite this, the effect of D614G might be confounded by other genetic variations among different virus isolates. For example, the D614G mutation was noted to be associated with mutations in viral nsp3 and an ORF1b protein variant (P314L) (5, 30). The coexistence of D614G and P314L may suggest additional mechanisms for the selection advantage of viruses expressing S-G614 in terms of viral infectivity and warrant further investigation.

In addition, our results showed quite interesting differences between the amounts of cleavage of S present in the supernatant (55.95% and 27.71%) and those in the lysates (64.96% and 39.13%). During biogenesis, the spike monomer travels from the endoplasmic reticulum (ER) to the ER-Golgi intermediate compartment (ERGIC), where it forms a trimer and incorporates into viral particles, budding into the lumens of vesicles, which are then trafficking for release through exocytosis (31). The cleavage of spike by active furin occurs primarily in the Golgi compartment and mainly in the trans-Golgi network (TGN) (32). The spike protein not packaged in the viruses remained in the membrane, which is more accessible for cleavage by the active furin in the TGN. Therefore, the cleavage form of spike, as reflected by the cleaved S/total S ratio, might be more predominant in the S from cell lysates than in that from the viruses in the supernatant. Based on the results, the difference in spike cleavage might be truly reflected by the analysis of the purified viruses. However, due to the safety concern, we did not conduct the virus purification experiment in the current study.

Moreover, we found a higher S/N ratio in S-G614 viruses than in S-D614 viruses (Fig. S2). Consistently, Zhang et al. reported higher spike density in S-G614 viruses (15). Through alignment of the closed conformation of the S-G614 trimer and S-D614 trimer by cryo-EM, Zhang et al. found that residues 620 to 640 at C-terminal domain 2 (CTD2) formed an ordered “630 loop” in the S-G614 trimer but a disordered one in the S-D614 trimer. The ordered loop might stabilize the cleaved S1/S2 trimer and prevent S1 shedding from S-G614 viruses (14), which provides a possible mechanism for the higher spike density in S-G614 viruses (15). Since the S1 subunit is responsible for the binding of S with ACE2, a decrease of S1 shedding from the trimer may increase the functional S protein for both viral infectivity and spike-mediated syncytium formation.

Currently, S-G614-containing SARS-CoV-2 has expanded and is the dominant strain worldwide. As the enhancement of cleavage by furin/PCs is critical for its transmission, blockade of this mechanism by targeting furin/PC protease activity for inhibition might become a potential antiviral strategy to block transmission of this strain. As shown in our recent study, two furin/PC inhibitors, CMK and naphthofluorescein, can abolish S cleavage, virus production, and pathogenic syncytium formation of an S-G614-containing SARS-CoV-2 isolate (NTU03) (17). Consistently, both CMK treatment and mutagenesis of the furin cleavage site can decrease syncytium formation in S-D614- and S-G614-expressing cells. The finding that CMK treatment can further decrease the luciferase reporter activity of the R682A mutant, suggesting that CMK might have some other effect on reporter activity, independently of the cleavage by the furin/PCs, needs further investigation. According to our studies, such furin inhibitors thus become leads for further antiviral development for the prevention and treatment of S-G614-containing SARS-CoV-2 infections.

## MATERIALS AND METHODS

### Viruses.

Sputum specimens from SARS-CoV-2-infected patients were kept in viral transport medium. Virus (multiplicity of infection [MOI] = 0.02) isolated from the specimens was propagated in Vero E6 cells in Dulbecco’s modified Eagle’s medium (DMEM) supplemented with 2 μg/ml tosylsulfonyl phenylalanyl chloromethyl ketone-trypsin, which may enhance infectivity and viral isolation efficacy, as documented previously (33–36). The 18 virus isolates used in the current study were hCoV-19/Taiwan/NTU01/2020 to hCoV-19/Taiwan/NTU18/2020.

### Plaque assay.

The plaque assay was performed as previously described, with minor modifications (37). In brief, the Vero E6 cells (2 × 10^5^ cells/well) were seeded in 24-well tissue culture plates and maintained in DMEM supplemented with 10% fetal bovine serum (FBS) and antibiotics. After 24 h of incubation, SARS-CoV-2 was treated to the cell monolayer for 1 h at 37°C. After removal of the virus, the cell monolayer was washed once with phosphate-buffered saline (PBS), and the cells were maintained with medium containing 1% methylcellulose and incubated for 5 to 7 days. Subsequently, cells were fixed with 10% formaldehyde overnight and stained with 0.5% crystal violet in order to count the plaques. Virus titers are means from three independent experiments.

### Plasmid construction.

Humanized pUC57-2019-nCoV-S was a kind gift from Che Ma at the Institute of Genomics Research Center, Academia Sinica, Taiwan. The spike sequence was cloned into the pcDNA3.0-HA vector with the addition of a hemagglutinin (HA) tag at the spike protein C terminus via NheI and XbaI sites. A QuikChange II site-directed mutagenesis kit (Agilent) was used to generate the mutant D614G spike or R682A spike construct. The primer set for D614G spike was S-D614G-F (5′-CTCGGTACAATTCACGCCCTGATACAGCACGGC-3′) and S-D614G-R (5′-GCCGTGCTGTATCAGGGCGTGAATTGTACCGAG-3′). The primer set for R682A spike was 5′-ACGCTCCGGGCTCTTGCGGGAGAGTTTGTCTG-3′ and 5′-CAGACAAACTCTCCCGCAAGAGCCCGGAGCGT-3′.

### Cell culture experiments.

293T cells stably expressing human ACE2 (293T-ACE2) were kindly provided by Mi-Hua Tao. The 293T, 293T-hACE2, and Vero E6 cells were maintained and grown at 37°C in DMEM (Biological Industries) containing 10% FBS (HyClone, GE Healthcare Life Sciences) in a 5% CO_2_ incubator. The Calu-3 cells were maintained in DMEM containing 15% FBS. The spike D614 or spike G614 plasmid was transfected into Vero E6 cells with Lipofectamine 2000 (Thermo Fisher Scientific). The furin inhibitor CMK (10 mM in dimethyl sulfoxide [DMSO]; final concentration, 50 μM in 0.5% DMSO [vol/vol]) (Tocris Bioscience) was added to the medium at the indicated concentrations 2 h posttransfection. Cells were harvested 24 h posttransfection for subsequent Western blotting.

### Cell-cell fusion assay.

A quantitative GAL4-based mammalian one-hybrid assay was established to assess cell-cell fusion activity. This reporter assay contains two plasmids (kindly provided by Hsiu-Ming Shih at the Institute of Biomedical Sciences, Academia Sinica, Taiwan). One is the reporter construct pGAL4/UAS-TK-Luc, which encodes firefly luciferase under the control of the GAL4 response element (upstream activation sequence [UAS]) and a thymidine kinase (TK) promoter. The other is the transcriptional activator construct pGAL4DBD-hAR-NTD, which consists of aa 1 to 560 of the AR transcriptional activation domain with the GAL4 DNA-binding domain (DBD) fused at its N terminus. The protein encoded by pGAL4DBD-hAR-NTD can bind to the UAS GAL4 response element of the pGAL4/UAS-TK-Luc reporter and activate the transcription of the luciferase reporter gene by the transcriptional activator of AR-NTD.

To detect cell-cell fusion activity, 293T-hACE2 and Vero E6 (which endogenously express ACE2) cells transfected with pGAL4/UAS-TK-Luc were prepared as target cells; 293T cells expressing the SARS-CoV-2 spike protein and pGAL4DBD-hAR-NTD were prepared as effector cells. In brief, 4 × 10^5^ 293T cells were seeded in 12-well plates, and Vero E6 and 293T-hACE2 cells were seeded in 24-well plates (2 × 10^5^/well) overnight. After 24 h, the 293T cells were cotransfected with the pGAL4DBD-hAR-NTD and D614-spike or G614-spike plasmid. Vero E6 cells and 293T-hACE2 cells were transfected with pGAL4/UAS-TK-Luc and pCMV-Renilla. Twenty-four hours posttransfection, 293T cells expressing the GAL4DBD-hAR-NTD protein and D614 spike or G614 spike protein were suspended in trypsin, and 1 × 10^5^ cells were seeded on Vero E6 or 293T-hACE2 cells expressing the GAL4/UAS-TK-Luc protein. The cells were cocultured for 24 h and harvested with passive lysis buffer (PLB) for the dual-luciferase assay according to the manufacturer’s instructions (Promega).

### Western blot analysis.

Western blotting was performed as previously described (38). In brief, cell lysates were extracted by 1× radioimmunoprecipitation assay (RIPA) buffer (Merck Millipore) containing 1× proteinase inhibitor (Merck Millipore) and 1× phosphatase inhibitor (Calbiochem). Equal amounts of protein samples were electrophoretically separated by 10% sodium dodecyl sulfate–polyacrylamide gel electrophoresis (SDS-PAGE) and transferred to polyvinylidene difluoride (PVDF) membranes. The membranes were probed with the indicated primary antibodies at 4°C overnight and then reacted with a secondary antibody. Antigen-antibody complexes were visualized using Western Lightning Plus-ECL (PerkinElmer). The antibodies used for Western blot analysis were as follows: rabbit anti-SARS-associated CoV (SCoV)/SARS-CoV-2 nucleocapsid (generated by our laboratory), mouse anti-SARS-CoV/SARS-CoV-2 (COVID-19) spike (1A9) (GeneTex; GTX632604), rabbit anti-glyceraldehyde-3-phosphate dehydrogenase (GAPDH) (GeneTex; GTX100118), horseradish peroxidase-conjugated mouse IgG (GeneTex; GTX213111-01), and rabbit IgG (GeneTex; GTX213110-01). The quantitative result of the ratio of cleaved to full-length spike in immunoblots was analyzed by VisionWorks Life Science Image Analysis software (UVP).

### RNA extraction and Northern blot analysis.

RNA was extracted with a NucleoSpin RNA kit (Macherey-Nagel) according to the manufacturer’s instructions. Northern blotting was performed as previously described (39). In brief, 0.2 μg of RNA was denatured and separated by an 0.8% agarose-formaldehyde gel at 70 V for 5 h. The agarose gel was soaked in 50 mM NaOH for 50 min to break the large RNA fragment. Subsequently, the gel was washed with 100 mM Tris-HCl (pH 7.5) for 30 min and incubated in 20× SSC buffer (1× SSC is 0.15 M NaCl plus 0.015 M sodium citrate) for 20 min and then capillary transferred to a positively charged Hybond-N nylon membrane (Amersham Biosciences) overnight. RNA was immobilized by UV cross-linking (1,800 × 100 μJ/cm) and hybridized at 50°C overnight with digoxigenin (DIG)-labeled probes generated with a PCR DIG probe synthesis kit (Roche Diagnostics). The primers used to synthesize the DIG-labeled nCoV19-N cDNA probe were 5′-AAGCTGGACTTCCCTATGGTGC-3′ and 5′-CCTTGGGTTTGTTCTGGACCACG-3′. The probes of porphobilinogen deaminase (PBGD) used as the internal control in Northern blotting were 5′-GGTGACCAGCACACTTTGGG-3′ and 5′-AGCCGGGTGTTGAGGTTTCC-3′.

### qRT-PCR.

The qRT-PCR was conducted by following the protocol described previously (17). In brief, RNA extracted from SARS-CoV-2-infected Vero E6 cells was reverse transcribed using a SuperScript III reverse transcriptase system (Thermo Fisher Scientific). Quantitative PCR targeting the E gene was performed using FastStart DNA SYBR green on a LightCycler 1.5 (Roche Diagnostics), with the primer set 5′-ACAGGTACGTTAATAGTTAATAGCGT-3′ and 5′-ATATTGCAGCAGTACGCACACA-3′. The RNA level of the E gene in the cells was determined relative to the internal control, the cellular PBGD gene, with the primer set 5′-GCATCGCTGAAAGGGCCTTCC-3′ and 5′-TCATCCTCAGGGCCATCTTCATGC-3′.

### Statistical analysis.

The virus titers quantified by plaque assays in triplicate are shown as means ± standard deviations (SD). Results from the reporter assay are shown as data representative of three independent experiments and are presented as means ± SD. Differences in data from the virus titer, qRT-PCR, and reporter assays between each indicated paired sample group were evaluated by Student's *t* test. A *P* value of 0.05 or lower was used to indicate statistical significance (*, *P < *0.05; **, *P < *0.01; ***, *P < *0.001).

### Data availability.

The sequencing data for the 18 virus isolates used in the current study (hCoV-19/Taiwan/NTU01/2020 to hCoV-19/Taiwan/NTU18/2020) have been deposited in GISAID (https://www.gisaid.org/CoV2020/). The accession codes are listed in Table S1 in the supplemental material, and the mutations in these 18 viral isolates are summarized in Table S2.
