# Supplementary figures and images for: D614G Substitution of SARS-CoV-2 Spike Protein Increases Syncytium Formation and Virus Titer via Enhanced Furin-Mediated Spike Cleavage
Source: mBio. 2021 Jul 27;12(4):e00587-21. doi: 10.1128/mBio.00587-21 (PMC8406174; doi:10.1128/mBio.00587-21)

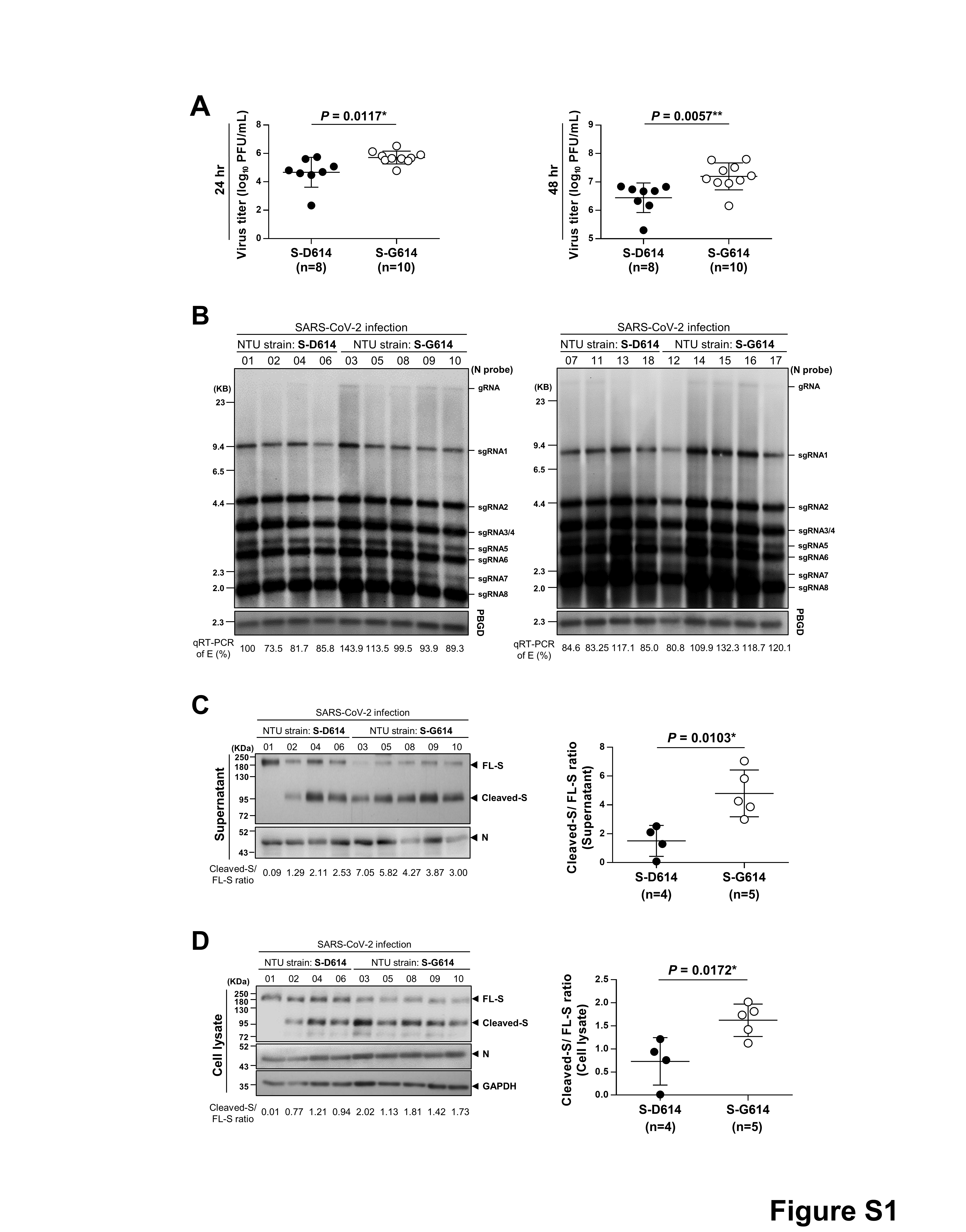

Supplement: FIG S1 [file mbio.00587-21-sf001.tif]

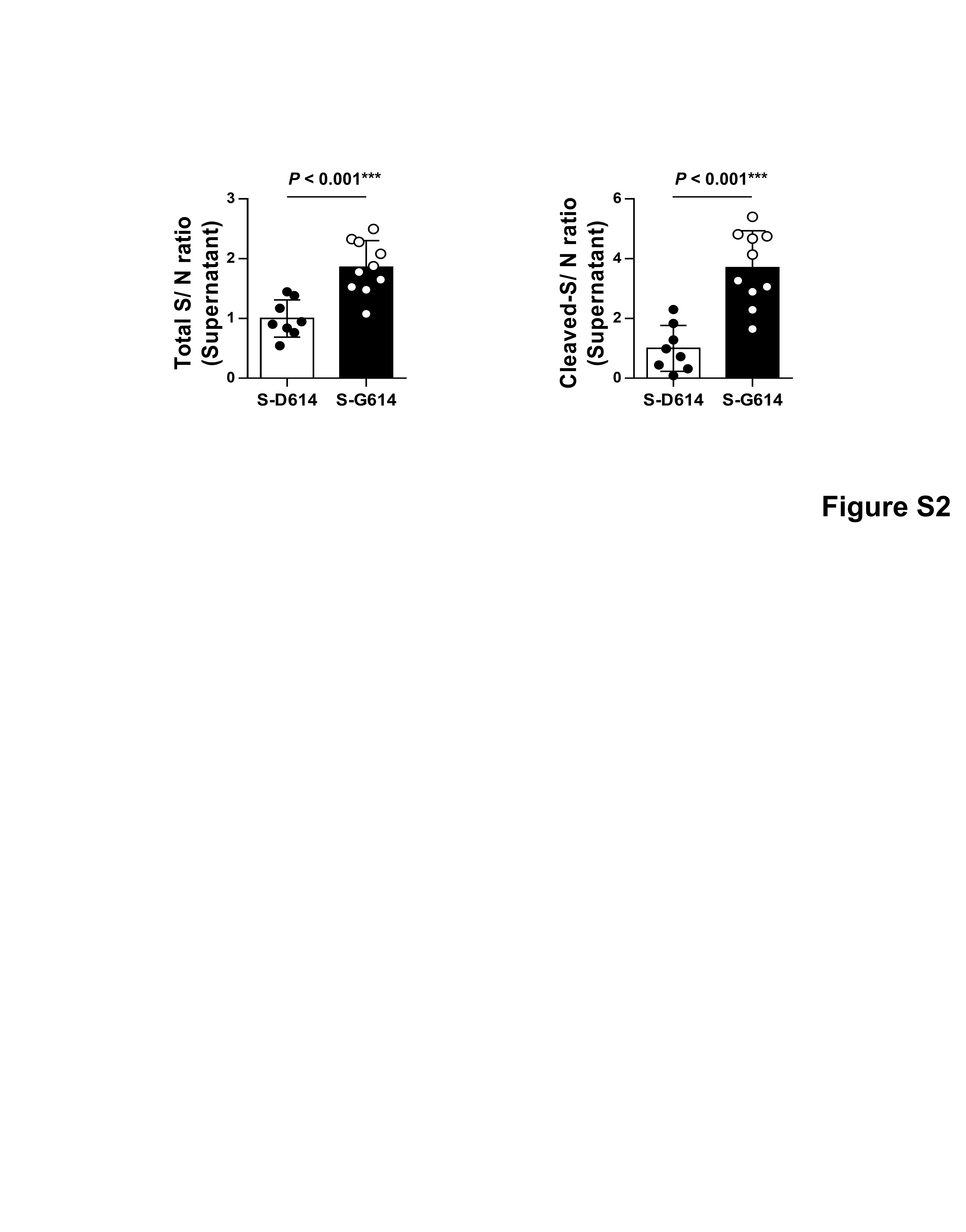

Supplement: FIG S2 [file mbio.00587-21-sf002.tif]
