# Supplementary material for: D614G Substitution of SARS-CoV-2 Spike Protein Increases Syncytium Formation and Virus Titer via Enhanced Furin-Mediated Spike Cleavage
Source: mBio. 2021 Jul 27;12(4):e00587-21. doi: 10.1128/mBio.00587-21 (PMC8406174; doi:10.1128/mBio.00587-21)
Supplement: TABLE S1 [file mbio.00587-21-st001.docx]

**Table S1: SARS-CoV-2 genome (GISAID ID)**

| Virus name | Accession ID | Location |
| --- | --- | --- |
| hCoV-19/Taiwan/NTU01/2020 | EPI_ISL_408489 | Asia / Taiwan / Taipei |
| hCoV-19/Taiwan/NTU02/2020 | EPI_ISL_410218 | Asia / Taiwan / Taipei |
| hCoV-19/Taiwan/NTU03/2020 | EPI_ISL_413592 | Asia / Taiwan / Taipei |
| hCoV-19/Taiwan/NTU04/2020 | EPI_ISL_422407 | Asia / Taiwan / Taipei |
| hCoV-19/Taiwan/NTU05/2020 | EPI_ISL_422408 | Asia / Taiwan / Taipei |
| hCoV-19/Taiwan/NTU06/2020 | EPI_ISL_422409 | Asia / Taiwan / Taipei |
| hCoV-19/Taiwan/NTU07/2020 | EPI_ISL_422410 | Asia / Taiwan / Taipei |
| hCoV-19/Taiwan/NTU08/2020 | EPI_ISL_422411 | Asia / Taiwan / Taipei |
| hCoV-19/Taiwan/NTU09/2020 | EPI_ISL_422412 | Asia / Taiwan / Taipei |
| hCoV-19/Taiwan/NTU10/2020 | EPI_ISL_447614 | Asia / Taiwan / Taipei |
| hCoV-19/Taiwan/NTU11/2020 | EPI_ISL_422413 | Asia / Taiwan / Taipei |
| hCoV-19/Taiwan/NTU12/2020 | EPI_ISL_422414 | Asia / Taiwan / Taipei |
| hCoV-19/Taiwan/NTU13/2020 | EPI_ISL_422415 | Asia / Taiwan / Taipei |
| hCoV-19/Taiwan/NTU14/2020 | EPI_ISL_422416 | Asia / Taiwan / Taipei |
| hCoV-19/Taiwan/NTU15/2020 | EPI_ISL_422417 | Asia / Taiwan / Taipei |
| hCoV-19/Taiwan/NTU16/2020 | EPI_ISL_422418 | Asia / Taiwan / Taipei |
| hCoV-19/Taiwan/NTU17/2020 | EPI_ISL_422419 | Asia / Taiwan / Taipei |
| hCoV-19/Taiwan/NTU18/2020 | EPI_ISL_447615 | Asia / Taiwan / Taipei |

The sequencing data and the accession codes of 18 virus isolates were deposited in the GISAID (https://www.gisaid.org/CoV2020/).
