# Supplementary material for: D614G Substitution of SARS-CoV-2 Spike Protein Increases Syncytium Formation and Virus Titer via Enhanced Furin-Mediated Spike Cleavage
Source: mBio. 2021 Jul 27;12(4):e00587-21. doi: 10.1128/mBio.00587-21 (PMC8406174; doi:10.1128/mBio.00587-21)
Supplement: TABLE S2 [file mbio.00587-21-st002.docx]

**Table S2. Summary of the mutations in SARS-CoV-2 isolates NTU01-NTU18.**

SARS-CoV-2 genetic sequences, including NTU01-NTU18 and the reference sequence (MN_908947), were aligned and compared. The nucleotide 23403, corresponding to the a.a. 614 of spike protein, was marked in red text, and the orange shaded nucleotides indicated nucleotides different from the reference nucleotide. Sequence data was obtained from GISAID (https://www.gisaid.org/CoV2020/); the GISAID accession numbers for these 18 isolates are listed in Supplementary Table S1.

Table S2. Summary of SARS-CoV-2 isolates NTU1-NTU18 alignment.
